# Removal and Biodegradation of Nonylphenol by Four Freshwater Microalgae

**DOI:** 10.3390/ijerph13121239

**Published:** 2016-12-14

**Authors:** Ning He, Xian Sun, Yu Zhong, Kaifeng Sun, Weijie Liu, Shunshan Duan

**Affiliations:** 1 Institute of Hydrobiology, Jinan University, Guangzhou 510632, China; hening2010@163.com (N.H.); imytian@163.com (X.S.); zhongyu@pku.edu.cn (Y.Z.); liuweijie_84@163.com (W.L.); 2 Key Laboratory of Aquatic Eutrophication and Control of Harmful Algal Blooms, Guangdong Higher Education Institutes, Guangzhou 510632, China; 3 Research Center of Offshore Marine Environment, South China Institute of Environmental Sciences, MEP, Guangzhou 510655, China; jnuskf@163.com

**Keywords:** nonylphenol, microalgae, removal, biodegradation, removal mechanism

## Abstract

The removal and biodegradation of nonylphenol (NP) by four freshwater microalgae, including three green algae (*Scendesmus quadriauda*, *Chlorella vulgaris*, and *Ankistrodesmus acicularis*) and one cyanobacterium (*Chroococcus minutus*) were studied in bacteria-free cultures exposed to different concentrations of NP for 5 days. All four algal species showed a rapid and high ability to remove NP (including bioaccumulation and biodegradation). Among these species, *A. acicularis* (*Ankistrodesmus acicularis*) had the highest NP removal rate (83.77%) at 120 h when exposed to different NP treatments (0.5–2.5 mg·L^−1^), followed by *C. vulgaris* (*Chlorella vulgaris*) (80.80%), *S. quadriauda (Scendesmus quadriauda)* (70.96%) and *C. minutus* (*Chroococcus minutus*) (64.26%). *C. vulgaris* had the highest NP biodegradation percentage (68.80%) at 120 h, followed by *A. acicularis* (65.63%), *S. quadriauda* (63.10%); and *C. minutus* (34.91%). The extracellular NP contents were lower than the intracellular NP contents in all tested algae. The ratio of the extracellular NP content and the intracellular NP content ranged from 0.04 to 0.85. Therefore, the removal of NP from the medium was mainly due to the algal degradation. These results indicate that *A. acicularis* and *C. vulgaris* are more tolerant to NP and could be used for treatment of NP contaminated aqueous systems effectively by bioremoval and biodegradation.

## 1. Introduction

Nonylphenol (NP) is a degradation product of the alkylphenol polyethoxylates (APEOs), an important class of nonionic surfactants employed in many detergent formulations for industrial and household use [1,2]. In the environment NP is both highly persistent and highly toxic, posing a serious threat to humans and other organisms due to its estrogenic properties [3,4]. Concentrations of 4-nonylphenol (NPs) as high as 325 μg·L^−1^ in surface water and up to 72 mg·kg^−1^ in sediment have been determined [5]. Conventional and advanced wastewater treatment methods are inefficient in removing NP [6,7], so new, cost-effective methods are needed to effectively remove NP from the contaminated environment.

Bioaccumulation and biodegradation of organic contaminants and even toxic pollutants has been reported. Those pollutants could be transformed into useful nutrients and growth-supporting substances by certain species of microalgae [8,9,10,11]. Green microalgae, such as *Chlorella vulgaris* and other *Chlorella* species have been used to remove organic matters, inorganic nutrients, heavy metals and even toxic organic contaminants from wastewater with both low cost and high efficiency [12,13,14,15]. Biotransformation of low-molecular weight phenols was reported in numerous species of microalgae, such as those isolated from olive-oil mill wastewaters [16]. Algae can have different interactions with aquatic contaminants, which include negative effects on algal growth and function and algal degradation of the contaminants. Growth of *Scenedesmus obliquus* was not affected by low NP concentrations (<1 mg·L^−1^), whereas algal growth was suppressed under high NP concentrations (>1 mg·L^−1^). In addition, more than 89% NP was removed by *S. obliquus* (*Scenedesmus obliquus*) due to biodegradation or biotransformation rather than the simple sorption on algal cell surface [17].

Reports have shown that species of microalgae harbor an attached bacterial flora, and also that various free-living bacteria coexist in algal cultures maintained in the laboratory [18,19]. These bacteria are an inherent part of the physical environment of algae and thus can be considered as symbionts [20,21]. Cultures of *Alexandrium tamarense*, as well as other dinoflagellates, often contain a considerable amount of bacteria from the original samples. These bacteria can produce substances which were either stimulatory or inhibitory to algae and thus change some characteristics of the algae, such as toxin production [22,23,24]. The elimination of bacteria did not affect the growth and toxin profile of *Alexandrium lusitanicum* and *A. tamarense* (*Alexandrium tamarense*), but it did affect the amount of toxins [25].

The bacterial communities of algal cultures interact with the algae, and thus, removal of bacteria from stock algal cultures would provide a simpler system for the study of NP removal and biodegradation by microalgae. The present study aimed to investigate the toxic effect of NP on *Scendesmus quadriauda* JNU39, *Ankistrodesmus acicularis* JNU14, *Chlorella vulgaris* JNU38 and *Chroococcus minutus* JNU17, and to evaluate the tolerance ability of the four different local freshwater microalgae to NP. The study also attempted to compare the NP removal and biodegradation ability among the four microalgae, and to identify the most effective species in an axenic medium. *S. quadriauda*, *A. acicularis* (*Ankistrodesmus acicularis*), *C. vulgaris* (*Chlorella vulgaris*) and *C. minutus* (*Chroococcus minutus*) are four microalgal species commonly used for the removal of wastewater-borne pollutants and they have a tolerance to some common pollutants found in wastewater, such as heavy metals, crude oil and polycyclic aromatic hydrocarbons [26,27,28,29].

## 2. Materials and Methods

### 2.1. Microalgal Species and Culture Conditions

Four freshwater microalgae, recorded as *S. quadriauda* JNU39, *A. acicularis* JNU14, *C. vulgaris* JNU38 and *C. minutus* JNU17, were tested in the present study. These species were isolated from NP-polluted water in Jinan University, Guangzhou, China. *S. quadriauda* was quadrate in shape with a dimension of 6.90 × 2.59 × 2.49 μm. *A. acicularis* was cylindrical in shape with diameter 2 μm and length 40 μm. *C. vulgaris* and *C. minutus* were spherical in shapes with diameters of 5.89 and 2.75 μm, respectively.

The microalgal culture of each species was cultivated in 2 L conical flasks containing 1000 mL BG11 medium, in an environmental chamber illuminated with cool white fluorescent tubes at a light intensity of 90 μmol·m^−2^·s^−1^, a diurnal cycle of 12 h light and 12 h dark and at a temperature of 25 ± 2 °C. Flasks were continuously shaken at 100 rpm. The components of basal culture medium were as follows: NaNO_3_ 1.5 g·L^−1^, K_2_HPO_4_ 40 mg·L^−1^, MgSO_4_·7H_2_O 75 mg·L^−1^, CaCl_2_·2H_2_O 36 mg·L^−1^, NaHCO_3_ 20 mg·L^−1^, ferric ammonium citrate 6 mg·L^−1^, citric acid 6 mg·L^−1^. The trace metal solution contained: H_3_BO_3_ 2.86 mg·L^−1^, MnCl_2_·4H_2_O 1.81 mg·L^−1^, ZnSO_4_·7H_2_O 222 mg·L^−1^, Na_2_MoO_4_·2H_2_O 390 mg·L^−1^, CuSO_4_·5H_2_O 79 mg·L^−1^, Co(NO_3_)_2_·6H_2_O 49.4 mg·L^−1^ [30].

### 2.2. Removal of Bacteria from Algal Cultures

Mid-exponential phase algal cultures (100 mL) were filtered through a 10 μm pore size membrane and subjected to the following treatments: the algal cells were suspended in 50 mL sterile BG11 medium before sequential centrifuging (1000× *g*, 10 min) and washing three times. The washed cells were suspended in 50 mL sterile BG11 medium containing 0.005% Tween-80 and 0.1 M EDTA (at 20 °C for 1 h) before lysozyme (0.5 mg·mL^−1^, 20 °C for 10 min) and SDS (Sodium Dodecyl Sulfate) (0.25%, 20 °C for 10 min) were added sequentially. The algal cells were centrifuged and washed twice to remove lysozyme and SDS and then resuspended in 50 mL sterile BG11 medium. The antibiotic cocktail containing 100 μg·mL^−1^ penicillin and 50 μg·mL^−1^ kanamycin were added to the treated algal cultures followed by incubation at 20 °C with a 12:12 light-dark cycle for 7 days. Assessment for bacterial presence was carried out after subculturing three times [31].

The algae were subjected to repeated washing, lysozyme/SDS and antibiotic treatment with a mixture of gentamycin, streptomycin, cephalothin and rifampicin. Axenic status was confirmed after subculturing three times in sterile BG11 medium without antibiotics. Bacteria could not be detected in the various media, both solid and liquid, nor by epifluorescence microscopy of both eubacteria and archaea. Bacterial presence was monitored throughout a full growth cycle and, following subculture, no bacteria were detected using the above methods [32].

### 2.3. NP Treatments

NP, purchased from Sigma-Aldrich (St. Louis, MO, USA) was dissolved in methanol as the stock solution at a concentration of 1 mg·mL^−1^. Microalgae cultures in the middle of the log phase of growth were decanted into 100 mL flasks containing 40 mL of medium at 25 ± 2 °C and illuminated with fluorescent lights (90 μmol·m^−2^·s^−1^ photon flux intensity) under a 12:12 h light/dark photoperiod. The cultures were initiated at 70.0 μg·L^−1^ chlorophyll a content, shaken periodically and used in triplicate. All solutions and experimental containers were autoclaved at 121 °C for 15 min. NP was added to the medium before inoculation at a concentration of 0, 0.5, 1.0, 1.5, 2.0 and 2.5 mg·L^−1^. Treatment with an equivalent amount of methanol (0.1%) was included as a control. The test lasted for 120 h.

### 2.4. Growth Analysis

The concentration of chlorophyll a in vivo was measured by TD-700 fluorometer (Turner Design, San Jose, CA, USA) every 24 h, which was calibrated with the standard solution of chlorophyll a. Prior to the detection, the test tubes were treated with dark adaption for 20 min at room temperature and shaken homogeneously for several times before determination. Content of chlorophyll a was measured using the excitation and emission wave lengths at 420 and 680 nm, respectively. Specific growth rate (μ) was calculated according to the following Equation (1):

μ = (lnX_t_ − lnX_0_)/(T_t_ − T_0_) (1)

where X_t_ and X_0_ are Chl a contents at times T_t_ and T_0_, respectively.

Optical density (OD) of the algae cultures was measured daily at 680 nm as the cell density and dry weight indicator using a BMG microplate reader (BMG Lab Technologies, Offenburg, Germany). The cell density was determined using a haemocytometer (Marienfeld, Lauda-Königshofen, Germany) under a light microscope. For cell dry weight measurement, a 20 mL aliquot of culture was filtered through pre-weighed 0.45 mm pore-size GF/F glass-fiber filter paper (Whatman, Maidstone, UK). The filter paper with algal cells was dried overnight in an oven at 60 °C till a constant weight was reached. The difference between the final weight and the weight before filtration was the dry weight of algal cells. The linear relationship between algal density (N, cells·mL^−1^), dry weight and OD_680_ is shown in the following equations:

JNU14 Cell density (10^4^ cells·mL^−1^) = 737.22 × OD_680_ + 3.6148 R^2^ = 0.989 (2)

JNU17 Cell density (10^4^ cells·mL^−1^) = 1013 × OD_680_ + 37.112 R^2^ = 0.9711 (3)

JNU38 Cell density (10^4^ cells·mL^−1^) = 450.28 × OD_680_ + 1.5385 R^2^ = 0.9951 (4)

JNU39 Cell density (10^4^ cells·mL^−1^) = 342.7 × OD_680_ + 7.4226 R^2^ = 0.9997 (5)

JNU14 Dry weight (g·L^−1^) = 0.9823 × OD_680_ + 0.0785 R^2^ = 0.9057 (6)

JNU17 Dry weight (g·L^−1^) = 1.3729 × OD_680_ + 0.0063 R^2^ = 0.9987 (7)

JNU38 Dry weight (g·L^−1^) = 1.0546 × OD_680_ − 0.0044 R^2^ = 0.9919 (8)

JNU39 Dry weight (g·L^−1^) = 1.4771 × OD_680_ − 0.0101 R^2^ = 0.9998 (9)

### 2.5. Determination of Residual NP

#### 2.5.1. NP Concentration Dissolved in the Medium

At each sampling timepoint, 5 mL cultures were withdrawn from the flasks and cells were separated from the culture through centrifugation at 4500 *g* for 15 min at 4 °C. The supernatant was extracted with liquid-liquid microextraction (DLLME), as described by Rezaee et al. [32], with some modifications. In brief, a 5 mL of sample was injected with 0.2 mL mixture of chlorobenzene and acetone (1:2) in a 10 mL screw cap glass test tube with a conical bottom. After gently shaking, a milky cloudy solution (water/chlorobenzene) was formed in the test tube. The sample was then centrifuged for 5 min at 4500 *g*. The dispersed fine particles of extraction phase which settled in the bottom of the conical test tube were withdrawn using a 50 μL microsyringe (zero dead volume, cone tip needle). This extraction process was repeated three times and the sediment fractions were combined for further analysis with high performance liquid chromatograph (HPLC) (Agilent, Santa Clara, CA, USA). All the extraction was performed at room temperature (23 ± 2 °C).

#### 2.5.2. NP Absorbed onto Cell Surface

The cell pellets from the above section were washed with 5 mL of 10 percent methanol and shaken for approximately 60 s, the NP contained in the water was considered as the surface adsorbed NP [33] and then extracted with DLLME, as described above and analyzed with HPLC.

#### 2.5.3. NP Concentration Absorbed into Cells

After adding appropriate amount of anhydrous Na_2_SO_4_, the cell pellets obtained from the above section were mixed with dichloromethane-methanol (1:2 v/v, 3 mL); after sonication for 20 min, the sample was centrifuged for 5 min at 3500 *g*. The cell pellets were extracted two more times and the solvent fractions were combined for further analysis with HPLC [34].

Based on the measured concentrations, the removal efficiency (R) and biodegradation percentage (BDP) of NP by the algal biomass were calculated as previously described [35] with minor modifications according to the following equations:

R = 100 × (C_i_ − C_f_)/C_i_ (10)

where R is the dissolved NP removal rate (percent); C_i_ and C_f_ are the initial and final concentrations (mg·L^−1^) of NP in the solution, respectively, and:

BDP (%) = 100 × (C_i_ − C_r_ − C_a_ − C_d_ × W_a_ − C_c_ × W_a_)/C_i_ (11)

where C_i_ is the initial concentration (mg·L^−1^) of NP in the solution, C_r_ is the residual concentration (mg·L^−1^) in the solution, C_a_ is the concentration of abiotic removal (mg·L^−1^), C_d_ is the concentration (mg·g^−1^) dry weight of NP adsorbed on the cell wall, C_c_ is the concentration (mg·g^−1^ dry weight) of NP accumulated in algal cells, and W_a_ is the dry weight of algal biomass expressed in g·L^−1^.

#### 2.5.4. Determination of NP

NP concentrations were analyzed by using an Agilent 1100 series high performance liquid chromatograph (HPLC) (Agilent, Santa Clara, CA, USA) coupled to a fluorescence detector. The elution was carried out under isocratic conditions with acetonitrile and Milli-Q water as the mobile phase. A XDB-C18 RS column (4.6 × 250 mm, 5 μm) was used and the volume ratio of acetonitrile to Milli-Q water was 80:20. The injection volume was 50 μL and the flow rate was set at 1 mL·min^−1^. The fluorescence detector was set at excitation and emission wavelengths of 230 and 305 nm, respectively. The retention time was 18 min. The limit of quantification for both NP was 5 μg·L^−1^. Results obtained here were compared to that obtained with a control group without nonylphenol.

### 2.6. Statistical Analysis

Statistical analysis was carried out using the SPSS16.0 package (SPSS Inc., Chicago, IL, USA). One Way-ANOVA followed by Tukey’s post hoc test was used to check the significance of treatments. Levels of significance used were 5% and 1%, described as “significant” and “highly significant”, respectively. Data are presented as mean ± standard deviation (mean ± SD) unless otherwise stated.

## 3. Results

### 3.1. Growth of Different Microalgae Species Exposed to NP

**Figure 1:**
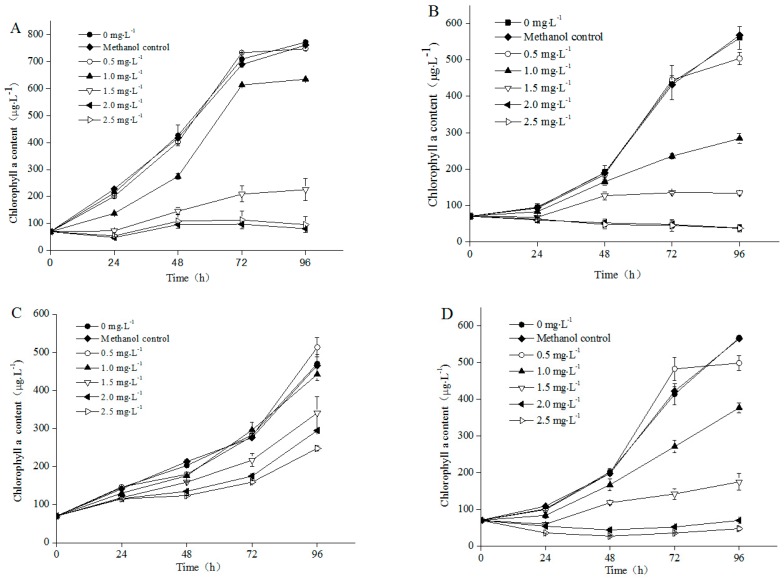
Effect of Nonylphenol (NP) concentration on the chlorophyll a content of (**A**) *A. acicularis*; (**B**) *C. minutus*; (**C**) *C. vulgaris* and (**D**) *S. quadriauda*, Values are the mean ± standard deviation (SD) (*n* = 3).

**Figure 2:**
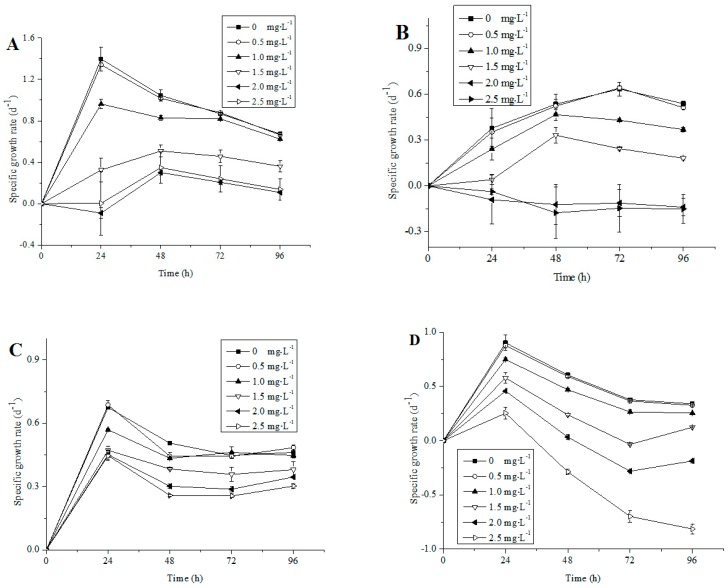
Growth of (**A**) *A. acicularis*; (**B**) *C. minutus*; (**C**) *C. vulgaris* and (**D**) *S. quadriauda*, exposed to different NP concentrations at the end of 0, 24, 48, 72 and 96 h (mean and standard deviation of three replicates were shown).

The chlorophyll a contents of the four nontoxic freshwater microalgae strains were influenced by NP (Figure 1). The solvent (methanol) in the designated concentration in the study (0.1%) had no obvious effect on algal growth (Figure 1). A significant decrease in growth, in terms of chlorophyll a concentration was observed in all four species when exposed to 1.5–2.5 mg·L^−1^ NP, as compared with their corresponding control cultures (Figure 1). The specific growth rate of four microalgae were not different with control under lower NP concentrations (<0.5 mg·L^−1^) (*p >* 0.05), whereas significant growth inhibition was determined under higher NP concentrations (1–2.5 mg·L^−1^) (Figure 2). The growth patterns of *A. acicularis*, *C. vulgaris* and *S. quadriauda* were similar under low NP concentration (0.5–1 mg·L^−1^) exposure, however, significant differences were observed under high NP concentrations exposure (1.5–2.5 mg·L^−1^) (Figure 2A,C,D). In addition, the inhibitory effects were heightened with increasing concentrations of NP during exposure time. At 96 h of culturing, the specific growth rate of the four species decreased with increasing concentrations of NP. A total of 2.5 mg·L^−1^ NP completely inhibited cell growth of the *C. minutus* and *S. quadriauda* (Figure 1B,D and Figure 2B,D).

Our experimental results have shown that at more than 0.5 mg·L^−1^ NP, any of the four algae was inhibited. However, *A. acicularis* and *C. vulgaris* have a high tolerance to NP (0.5–1 mg·L^−1^, Figure 2A,C). Among four species, the growth of *A. acicularis* and *C. vulgaris* completely recovered to the control level at 96 h, whereas the growth of *C. minutus* and *S. quadriauda* was significantly inhibited at the end of the experiment (Figure 1B,D), when compared to that of their corresponding control, suggesting that *A. acicularis* and *C. vulgaris* were the most adaptive species to 0–1 mg·L^−1^ NP concentration from the four test algae.

### 3.2. Removal of NP by Different Microalgae Species and Its Mechanisms

The residual concentrations of NP in the medium in the control flasks (without microalgal inoculation) did not show any significant changes during the 120 h experiments (data not shown), indicating that abiotic loss was negligible. The amount of NP remaining in the medium inoculated with microalgae species all decreased substantially within the first 24 h (Table 1). The decrease of NP was in a slower process, especially after a 72 h exposure (Table 2 and Table 3). The contents of NP in four algal cells under different treatments were measured (Table 1, Table 2 and Table 3) and it was observed that from the extra/intra ratios shown in Table 1, Table 2 and Table 3, the extracellular NP contents in all four species have been lower than the intracellular NP contents, with the ratios changing from 0.04 to 0.85.

**Figure 3:**
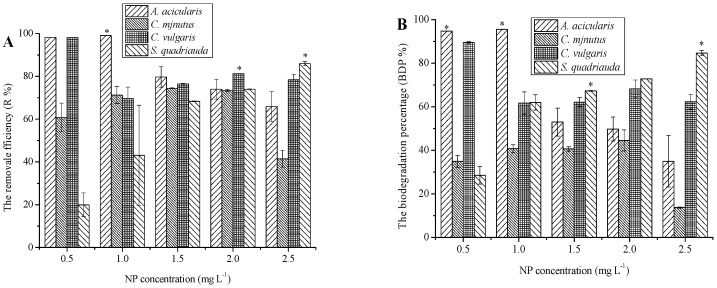
Removal (**A**) and biodegradation (**B**) of NP by *A. acicularis*, *C. minutus*, *C. vulgaris* and *S. quadriauda* at the end 120 h (mean and standard deviation of three replicates are shown. The single asterisk * in the figure represents significant difference compared to other algae species (*p <* 0.05).

As the results in Table 1, Table 2 and Table 3 demonstrated, biodegradation process was responsible for the removal of NP. The biodegradation percentages of NP by species decreased, whereas the removal efficiency percentages decreased with increasing NP bioconcentration (from 0.5 to 2.5 mg·L^−1^). Among all species, *A. acicularis* was the most effective species and removed more than 90% NP at low NP concentration exposure (0.5–1.0 mg·L^−1^) (Figure 3A). The process of removed NP by *A. acicularis* was mainly caused by biodegradation (Figure 3B, Table 3). At more than 1.5 mg·L^−1^ NP concentration, the specific growth rate and removal efficiency were decreased significantly (*p* < 0.05). Further, more than 90% of NP was removed by *C. vulgaris* at the end of the experiment under the 0.5 mg·L^−1^ NP treatment (Figure 3A). When the four species were exposed to 2.0–2.5 mg·L^−1^ NP, *C. vulgaris* and *S. quadriauda* removed more than 80% NP. For *S. quadriauda*, the NP degradation efficiency increased gradually with increasing concentrations of NP. However the NP degradation ability of three species decreased with increasing NP concentrations (Figure 3B). More than 60% of NP removed by *S. quadriauda* were attributed to biodegradation processes at 2.0–2.5 mg·L^−1^ NP. However, the growth of *S. quadriauda* was inhibited at more than 2.0 mg·L^−1^ NP. We supposed that some of the products by biodegradation may inhibit *S. quadriauda* growth. We were keeping research the specific biodegradation mechanism of *S. quadriauda*. In contrast, *C. minutus* displayed not only the lowest NP degradation ability, but also a more or less constant NP degradation efficiency during the experiment, with only 15%–40% NP biodegraded at the end of the experiment under different NP concentrations (Figure 3B). The highest of NP removed by *C. minutus* was 65% at the end of the experiment under the 1.5–2.0 mg·L^−1^ NP treatment. At more than 2.0 mg·L^−1^ NP concentration, the specific growth rate and removal efficiency of *C. minutus* were decrease significantly (*p* < 0.05) (Figure 1, Figure 2 and Figure 3).

## 4. Discussion

### 4.1. Influence of NP on Algal Growth

As an inherent part of the physical environment of dinoflagellates, bacteria could exist in the medium, attached to algal cell walls or even within the algal cells. This results in great difficulty in obtaining axenic cultures. A variety of procedures have been used to obtain bacteria-free algal cultures for the study of the relationship between toxic organic contaminants and algae [36]. The growth curves of the algae in the present study indicated that all algal cells were on the logarithmic phase of growth. When NP concentration was increased, growth of algal cells was inhibited in different degrees and the growth curves presented dose-effect relationship. The NP effect on growth inhibition in four algae (Figure 1) was in agreement with earlier reports [36,37] for freshwater algae, where a concentration of 500 μg·L^−1^ NP was able to reduce algal growth (EC_50_).

The present study clearly showed that the NP concentrations lower than 0.5 mg·L^−1^ did not markedly affect the algal growth of the four microalgae species, but when the concentrations were higher than 1.5 mg·L^−1^, the growth of the algal species decreased. A similar cell response was also observed in *Microcystis aeruginosa* and *Chlorella* species after NP exposure [38,39,40], and *Chlorella fusca*, *Monoraphidium braunii* and *Stephanodiscus hantzschii* after bisphenol-A (BPA) exposure [41,42,43]. The chlorophyll a curves showed that the *A. acicularis*, *C. minutus* and *S. quadriauda* had negative growth under 2.0–2.5 mg·L^−1^ NP exposure, whereas the *C. vulgaris* still grew well (Figure 1). Therefore, *C. vulgaris* were more resistant to NP than the other species at concentrations above 2 mg·L^−1^.

Microalgae possess several mechanisms for protecting themselves from the toxicity of organic contaminants. The cell wall, composed mainly of carbohydrates and proteins, serves as a barrier between organic pollutants and the cell interior [44,45]. In the present study, *C. vulgaris* might formed thicker cell walls than the other species to improve their adaptation to the stress caused by high concentrations of target compounds, and above results proved in some previous studies on this species exposed by the heavy metals zinc and copper [33,39,44,46]. Tsang et al. reported that *C. vulgaris* resisted tributyltin (TBT) by transforming TBT into a much less toxic metabolite [44]. Some microalgae species such as *Chlorella fusca* could metabolize BPA to an intermediate with no estrogenic activity [47]. Probably the phenolic organics were utilized by the algae as carbon source and assimilated by cell components for cell growth. No intermediates of NP were detected in the present study, metabolic product and its mechanism deserves further study.

### 4.2. Capacity of Algae for the Removal of Contaminants

The capability of freshwater micro- and macro-algae to adsorb pollutants was highly dependent on the cell biovolume and surface area, in particular, the ratio of surface area to volume [48]. However, the relationship between the amounts of NP uptake and the surface area/volume ratio in the present study was insignificant, as the NP removal efficiency among four algae species of different sizes and shapes were comparable. Tsezos and Bell found that the toxic organic pollutants removal capacity of the cell walls was less than that of the cell contents [49]. These results suggested that in addition to cell volume and shape, other properties, such as composition and structure of the cell, might also be important in determining NP biosorption. In this study, most of the NP accumulated inside the cells (74%–87%), whereas only 13%–26% was adsorbed by the cell walls. These high values were comparable to that reported for macroalgae, such as *Cladophora* [6], and were much higher than that for a marine microalga, *Isochrysis galbana* [34]. Due to the little accumulation of NP in the algal cells (Table 1, Table 2 and Table 3), the results from the present study showed that the removal of NP by algae was mainly caused by biodegradation by the algal cells rather than by simple sorption and accumulation in the cells. This is consistent with the results of previous studies on the BPA removal by some microalgae species [43,50], NP removal by *C. vulgaris* and *Selenastrum capricornutum* [39]. After adsorption and absorption, the target compounds NP were first accumulated and then metabolized by algal cells; thus the amounts of NP accumulated in algal cells were much less than the amounts biodegraded. Several freshwater microalgae were found to be able to glycosylate BPA by the action of glycosyl transferase [47]. *S. obliquus* might have a similar mechanism to metabolize NP because these two compounds also possess the requisite functional group (–OH) for direct metabolism. 

Biodegradation of organic contaminants by algae has been demonstrated in previous and present studies. Tributyltin could be biodegradation by two *Chlorella* species [44] and some algae (such as *Anabaena flosaquae* and *Microcysis aeruginosa*) even were capable of producing di(*n*-butyl)phthalate (DBP) or mono(2-ethylhexyl)phthalate (MEHP) or both [51]. In the present study, NP showed an initial rapid removal phase during the first 24 h, followed by a slow dissipation phase (Table S1). Several processes might be involved in the dissipation processes, including sorption and biodegradation. It is expected that photolysis of NP induced by the presence of algae might occur, as demonstrated for the enhancement of BPA photodegradation by *C. vulgaris* and *Anabaena cylindrical* [52]. However, Table S1 clearly showed little variation in NP concentrations in the controls within 5 days. Therefore, the photolysis process played a small role in the dissipation of NP.

After the adsorption and absorption processes, NP was gradually degraded with 65.63%, 34.91%, 68.80% and 63.10% of the spiked NP degraded by *A. acicularis*, *C. minutus*, *C. vulgaris* and *S. quadriauda* at the end of the 120 h exposure under NP concentrations (0.5–2.5 mg·L^−1^), respectively. Such *A. acicularis*, *C. vulgaris* and *S. quadriauda* biodegradation was much faster than the previous results from two *M. aeruginosa* strains, which exhibited more than 60% NP degradation after 12 days of incubation, with different concentrations of NP [38] and microalgal species from other taxa [34]. These findings suggested that potential use of *A. acicularis*, *C. vulgaris* and *S. quadriauda* in the treatment of waste water containing the compound. The algae species *A. acicularis*, *C. vulgaris* and *S. quadriauda* used in the present study had nearly an equal removal capacity for NP as the green microalgae *Selenastrum capricornutum* and *Chlorella* species [39]. Therefore, algae used in the waste water treatment could not only remove heavy metals and inorganic substances such as nitrogen and phosphorus [46], but also remove organic substances such as NP [15,17,53,54]. *Chlorella* species, especially the commercial species *C. vulgaris*, were more capable of degrading NP than other algal genera, and the mechanisms involved two processes, a rapid initial passive physiochemical adsorption followed by active absorption, accumulation and degradation process.

The NP biodegradation pathway has been widely investigated in bacteria, but less so in plants, particularly in microalgae. Nevertheless, the metabolism of other phenolic compounds by microalgae displayed similar patterns as that in higher plants. Different freshwater microalgae were found to metabolize BPA to BPA glycosides, which were then released into the culture medium [47,55]. The metabolic pathway of *p*-chlorophenol (*p*-CP) in a marine microalga, *Tetraselmis marina*, involved glucosyl transfer followed by malonyl transfer [56]. The diatom *Skeletonema costatum* was able to detoxify 2,4-dichlorophenol by conjugation to glutathione catalyzed by glutathione S-transferase [57]. Further studies are needed to identify the major metabolic products and the biodegradation pathways of NP by microalgae.

The present and previous studies all showed that the NP might subsequently pose potential risks to organisms at higher trophic levels via biomagnification along food chains in aquatic ecosystems. Although the concentrations of NP used in the present study are unlikely to be detected in aquatic ecosystems, the algae *A. acicularis*, *C. vulgaris* and *S. quadriauda* demonstrated a high capability for the removal of the NP at mg·L^−1^ levels, indicating good prospects for their use in the treatment of wastewater.

## 5. Conclusions

All the four microalgae species investigated in the present study could efficiently remove NP at a low concentration of 0.5–1 mg·L^−1^ from water (close to the highest NP concentration detected in the environment), within a short exposure time (within 24 h), under photoautotrophic conditions. The mechanisms included initial rapid adsorption and absorption, followed by accumulation and biodegradation. In addition, the removal by the four algae species was mainly attributed to biodegradation or biotransformation process by the algal cells rather than to simple sorption and accumulation in the cells. The amounts of NP adsorbed on the algal cells were lower than those absorbed in algal cells. The NP biodegradation ability was species-specific. Among four algae species, *A. acicularis* and *C. vulgaris* were the more suitable species for effective removal and biodegradation of NP, and potential application of microalgae species in the removal of organic contaminants including alkylphenols in addition to nutrients and metals.

## Figures and Tables

**Table 1:** Dissolved, intracellular and extracellular NP contents of four freshwater microalgae under different treatments at the end of 24 h. Mean and standard deviation of three replicates are shown.

Treatment (mg·L^−1^)	Microalgal Species	Amount of NP			Extra/Intra Ratio *
		Dissolved NP (μg·L^−1^)	Extracellular NP (10^−8^ μg·cell^−1^)	Intracellular NP (10^−8^ μg·cell^−1^)	
0.5	Control	445.0 ± 10.0	-	-	-
	*A. acicularis*	162 ± 0	0.9 ± 0.1	3.5 ± 0	0.26
	*C. minutus*	343.7 ± 1.2	1.0 ± 0.1	4.8 ± 0.7	0.21
	*C. vulgaris*	388.3 ± 11.7	0.8 ± 0.0	6.7 ± 0.6	0.12
	*S. quadriauda*	415.7 ± 32.0	9.7 ± 0.1	20.2 ± 2.8	0.49
1	Control	941.1 ± 7.2	-	-	-
	*A. acicularis*	405.9 ± 15.6	3.0 ± 0	14.1 ± 0.6	0.21
	*C. minutus*	515.9 ± 62.1	2.0 ± 0.2	14.5 ± 3.7	0.15
	*C. vulgaris*	723.7 ± 13.7	2.7 ± 0.1	9.6 ± 0.6	0.28
	*S. quadriauda*	770.2 ± 5.2	14.1 ± 3.2	37.2 ± 2.5	0.85
1.5	Control	1479.7 ± 10.2	-	-	-
	*A. acicularis*	665.3 ± 87.8	8.4 ± 0.8	37.0 ± 2.4	0.23
	*C. minutus*	964.9 ± 22.4	3.7 ± 0.6	24.8 ± 0.8	0.15
	*C. vulgaris*	1464.3 ± 154.8	8.1 ± 0.3	23.7 ± 2.9	0.35
	*S. quadriauda*	1477.8 ± 16.4	18.2 ± 0.6	61.7 ± 6.5	0.3
2	Control	1890 ± 13.0	-	-	-
	*A. acicularis*	845.6 ± 0.3	10.4 ± 0.2	49.8 ± 1.1	0.21
	*C. minutus*	1236.6 ± 41.6	4.9 ± 0.5	28.8 ± 2.6	0.17
	*C. vulgaris*	1305.2 ± 211.6	10.2 ± 1.0	33.4 ± 3.9	0.31
	*S. quadriauda*	1795.6 ± 132.1	23.2 ± 2.9	104.6 ± 0.6	0.22
2.5	Control	2401.0 ± 92.6	-	-	-
	*A. acicularis*	1360.5 ± 169.1	16.9 ± 1.2	77.0 ± 1.9	0.22
	*C. minutus*	1606.5 ± 136.2	6.6 ± 0.6	38.0 ± 0.5	0.16
	*C. vulgaris*	2308.5 ± 52.5	13.1 ± 1.4	38.3 ± 3.3	0.34
	*S. quadriauda*	1911.0 ± 8.7	50.3 ± 0.3	124.4 ± 1.3	0.4

* Ratio of extracellular concentration to intracellular concentration.

**Table 2:** Dissolved, intracellular and extracellular NP contents of four freshwater microalgae under different treatments at the end of 72 h. Mean and standard deviation of three replicates are shown.

Treatment (mg·L^−1^)	Microalgal Species	Amount of NP			Extra/Intra Ratio *
		Dissolved NP (μg·L^−1^)	Extracellular NP (10^−8^ μg·cell^−1^)	Intracellular NP (10^−8^ μg·cell^−1^)	
0.5	Control	448.3 ± 5.6	-	-	-
	*A. acicularis*	9.1 ± 0	0.2 ± 0	0.5 ± 0.1	0.33
	*C. minutus*	191.0 ± 0	0.1 ± 0	2.3 ± 0.3	0.06
	*C. vulgaris*	270.8 ± 3.4	1.0 ± 0.1	3.8 ± 0.6	0.26
	*S. quadriauda*	323.0 ± 0.9	1.7 ± 0.1	2.1 ± 0.2	0.85
1	Control	940.2 ± 10.1	-	-	-
	*A. acicularis*	117.0 ± 5.7	0.6 ± 0.1	1.9 ± 0.2	0.3
	*C. minutus*	402.4 ± 19.5	1.2 ± 0.1	7.1 ± 1.9	0.18
	*C. vulgaris*	549.1 ± 8.6	2.3 ± 0.2	7.8 ± 0.7	0.3
	*S. quadriauda*	478.1 ± 7.2	0.6 ± 0	2.7 ± 0.4	0.23
1.5	Control	1478.9 ± 23.6	-	-	-
	*A. acicularis*	539.3 ± 7.8	4.6 ± 0.7	19.2 ± 5.4	0.24
	*C. minutus*	760.1 ± 13.7	2.7 ± 0.1	16.5 ± 0.4	0.16
	*C. vulgaris*	840.6 ± 67.1	4.2 ± 0.4	18.7 ± 1.1	0.23
	*S. quadriauda*	720.2 ± 14.4	0.9 ± 0	4.0 ± 1.0	0.26
2	Control	1873.2 ± 26.0	-	-	-
	*A. acicularis*	599.3 ± 1.3	7.1 ± 0.2	24.1 ± 0.2	0.29
	*C. minutus*	1006.7 ± 29.1	3.6 ± 0.1	19.0 ± 2.1	0.18
	*C. vulgaris*	863.2 ± 45.2	5.5 ± 1.0	24.1 ± 4.1	0.23
	*S. quadriauda*	944.6 ± 21.6	1.9 ± 0.1	5.8 ± 0.6	0.32
2.5	Control	2399.6 ± 27.8	-	-	-
	*A. acicularis*	819.4 ± 31.7	11.3 ± 1.3	65.2 ± 10.0	0.17
	*C. minutus*	1429.4 ± 62.1	6.1 ± 0.2	27.7 ± 1.3	0.22
	*C. vulgaris*	1529.3 ± 46.4	7.2 ± 0.9	28.8 ± 3.1	0.25
	*S. quadriauda*	1391.2 ± 11.3	3.4 ± 0.5	12.8 ± 0.4	0.29

* Ratio of extracellular concentration to intracellular concentration.

**Table 3:** Dissolved, intracellular and extracellular NP contents of four freshwater microalgae under different treatments at the end of 120 h. Mean and standard deviation of three replicates are shown.

Treatment (mg·L^−1^)	Microalgal Species	Amount of NP			Extra/Intra Ratio *
		Dissolved NP (μg·L^−1^)	Extracellular NP (10^−8^ μg·cell^−1^)	Intracellular NP (10^−8^ μg·cell^−1^)	
0.5	Control	448.0 ± 9.1	-	-	-
	*A. acicularis*	9.1 ± 0	0.0 ± 0	0.4 ± 0	0.04
	*C. minutus*	196.1 ± 37.3	0.1 ± 0	1.5 ± 0.2	0.08
	*C. vulgaris*	9.1 ± 0	0.5 ± 0.0	2.6 ± 0	0.2
	*S. quadriauda*	200.0 ± 22.5	0.3 ± 0.0	0.1 ± 0	3.04
1	Control	943.9 ± 8.5	-	-	-
	*A. acicularis*	9.1 ± 0	0.2 ± 0	0.7 ± 0	0.3
	*C. minutus*	287.3 ± 32.4	0.1 ± 0	3.8 ± 0.9	0.04
	*C. vulgaris*	302.9 ± 42.8	1.7 ± 0.4	4.3 ± 0.9	0.4
	*S. quadriauda*	335.1 ± 19.1	0.4 ± 0	0.6 ± 0.1	0.6
1.5	Control	1475.9 ± 14.8	-	-	-
	*A. acicularis*	304.6 ± 60.5	3.9 ± 0.2	13.0 ± 0.2	0.3
	*C. minutus*	384.4 ± 2.2	0.7 ± 0.1	14.2 ± 0.3	0.05
	*C. vulgaris*	352.6 ± 2.3	3.6 ± 0.6	11.9 ± 2.2	0.31
	*S. quadriauda*	474.8 ± 2.7	0.4 ± 0	1.0 ± 0	0.42
2	Control	1880.5 ± 7.7	-	-	-
	*A. acicularis*	520.1 ± 75.9	4.8 ± 0.3	14.0 ± 0.4	0.34
	*C. minutus*	533.5 ± 7.8	1.1 ± 0.2	14.0 ± 1.2	0.08
	*C. vulgaris*	375.7 ± 0.7	3.1 ± 0.7	8.8 ± 2.6	0.37
	*S. quadriauda*	519.9 ± 1.8	0.7 ± 0	1.77 ± 0.2	0.4
2.5	Control	2397.9 ± 77.8	-	-	-
	*A. acicularis*	803.1 ± 102.9	6.5 ± 0.05	23.9 ± 3.6	0.27
	*C. minutus*	1465.3 ± 82.5	1.6 ± 0.4	19.6 ± 0.4	0.08
	*C. vulgaris*	539.9 ± 52.8	3.5 ± 0.7	12.6 ± 1.8	0.28
	*S. quadriauda*	351.6 ± 18.8	2.6 ± 0.2	5.7 ± 0.8	0.46

* Ratio of extracellular concentration to intracellular concentration.

## Supplementary Materials

The following are available online at www.mdpi.com/1660-4601/13/12/1239/s1, Table S1: The NP contents in medium of four freshwater microalgae under different treatments at 24, 72 and 120 h exposure.
