# Supplementary material for: Removal and Biodegradation of Nonylphenol by Four Freshwater Microalgae
Source: Int J Environ Res Public Health. 2016 Dec 14;13(12):1239. doi: 10.3390/ijerph13121239 (PMC5201380; doi:10.3390/ijerph13121239)
Supplement: Supplementary file 1 [file ijerph-13-01239-s001.pdf]

Supplementary Materials: Removal and Biodegradation of Nonylphenol by Four Freshwater Microalgae

Ning He, Xian Sun, Yu Zhong, Kaifeng Sun, Weijie Liu, Shunshan Duan

Table S1. The Nonylphenol (NP) contents in medium of four freshwater microalgae under different treatments at 24, 72 and 120 h exposure.

Treatment ($mg·L^{-1}$)	Microalgal Species	The Residual NP in the Medium($μg·L^{-1}$)			
		**0 h**	**24 h**	**72 h**	**120 h**
0.5	Control	447.1 ± 10.7	445.0 ± 10.0	448.3 ± 5.6	448.0 ± 9.1
	A. acicularis	-	162 ± 0	9.1 ± 0	9.1 ± 0
	C. mjnutus	-	343.7 ± 1.2	191.0 ± 0	196.1 ± 37.3
	C. vulgaris	-	388.3 ± 11.7	270.8 ± 3.4	9.1 ± 0
	S. quadriauda	-	415.7 ± 32.0	323.0 ± 0.9	200.0 ± 22.5
1	Control	951.2 ± 5.5	941.1 ± 7.2	940.2 ± 10.1	943.9 ± 8.5
	A. acicularis	-	405.9 ± 15.6	117.0 ± 5.7	9.1 ± 0
	C. mjnutus	-	515.9 ± 62.1	402.4 ± 19.5	287.3 ± 32.4
	C. vulgaris	-	723.7 ± 13.7	549.1 ± 8.6	302.9 ± 42.8
	S. quadriauda	-	770.2 ± 5.2	478.1 ± 7.2	335.1 ± 19.1
1.5	Control	1484.4 ± 12.1	1479.7 ± 10.2	1478.9 ± 23.6	1475.9 ± 14.8
	A. acicularis	-	665.3 ± 87.8	539.3 ± 7.8	304.6 ± 60.5
	C. mjnutus	-	964.9 ± 22.4	760.1 ± 13.7	384.4 ± 2.2
	C. vulgaris	-	1464.3 ± 154.8	840.6 ± 67.1	352.6 ± 2.3
	S. quadriauda	-	1477.8 ± 16.4	720.2 ± 14.4	474.8 ± 2.7
2	Control	1889.4 ± 20.0	1890 ± 13.0	1873.2 ± 26.0	1880.5 ± 7.7
	A. acicularis	-	845.6 ± 0.3	599.3 ± 1.3	520.1 ± 75.9
	C. mjnutus	-	1236.6 ± 41.6	1006.7 ± 29.1	533.5 ± 7.8
	C. vulgaris	-	1305.2 ± 211.6	863.2 ± 45.2	375.7 ± 0.7
	S. quadriauda	-	1795.6 ± 132.1	944.6 ± 21.6	519.9 ± 1.8
2.5	Control	2399.9 ± 59.3	2401.0 ± 92.6	2399.6 ± 27.8	2397.9 ± 77.8
	A. acicularis	-	1360.5 ± 169.1	819.4 ± 31.7	803.1 ± 102.9
	C. mjnutus	-	1606.5 ± 136.2	1429.4 ± 62.1	1465.3 ± 82.5
	C. vulgaris	-	2308.5 ± 52.5	1529.3 ± 46.4	539.9 ± 52.8
	S. quadriauda	-	1911.0 ± 8.7	1391.2 ± 11.3	351.6 ± 18.8

© 2016 by the authors; licensee MDPI, Basel, Switzerland. This article is an open access article distributed under the terms and conditions of the Creative Commons by Attribution (CC-BY) license (http://creativecommons.org/licenses/by/4.0/).
